# DNA Double Strand Break Response and Limited Repair Capacity in Mouse Elongated Spermatids

**DOI:** 10.3390/ijms161226214

**Published:** 2015-12-16

**Authors:** Emad A. Ahmed, Harry Scherthan, Dirk G. de Rooij

**Affiliations:** 1 Laboratory of Immunology and Molecular Physiology, Department of Zoology, Faculty of Science, Assiut University, Assiut 71516, Egypt; 2 Department of Molecular Biology and Biotechnology, University of Sheffield, Sheffield S10 2TN, UK; 3 Institute für Radiobiologie der Bundeswehr in Verb. mit der University, Ulm, Neuherbergstr, 11, Munich D-80937, Germany; scherth@web.de; 4 Reproductive Biology Group, Division of Developmental Biology, Department of Biology, Faculty of Science, Utrecht University, Utrecht 3584CM, The Netherlands

**Keywords:** DNA repair, Rad54/Rad54B deficient mice, SCID mice, PARP1-inhibited mice, elongated spermatids, NHEJ

## Abstract

Spermatids are extremely sensitive to genotoxic exposures since during spermiogenesis only error-prone non homologous end joining (NHEJ) repair pathways are available. Hence, genomic damage may accumulate in sperm and be transmitted to the zygote. Indirect, delayed DNA fragmentation and lesions associated with apoptotic-like processes have been observed during spermatid elongation, 27 days after irradiation. The proliferating spermatogonia and early meiotic prophase cells have been suggested to retain a memory of a radiation insult leading later to this delayed fragmentation. Here, we used meiotic spread preparations to localize phosphorylate histone H2 variant (γ-H2AX) foci marking DNA double strand breaks (DSBs) in elongated spermatids. This technique enabled us to determine the background level of DSB foci in elongated spermatids of RAD54/RAD54B double knockout (dko) mice, severe combined immunodeficiency SCID mice, and poly adenosine diphosphate (ADP)-ribose polymerase 1 (PARP1) inhibitor (DPQ)-treated mice to compare them with the appropriate wild type controls. The repair kinetics data and the protein expression patterns observed indicate that the conventional NHEJ repair pathway is not available for elongated spermatids to repair the programmed and the IR-induced DSBs, reflecting the limited repair capacity of these cells. However, although elongated spermatids express the proteins of the alternative NHEJ, PARP1-inhibition had no effect on the repair kinetics after IR, suggesting that DNA damage may be passed onto sperm. Finally, our genetic mutant analysis suggests that an incomplete or defective meiotic recombinational repair of Spo11-induced DSBs may lead to a carry-over of the DSB damage or induce a delayed nuclear fragmentation during the sensitive programmed chromatin remodeling occurring in elongated spermatids.

## 1. Introduction

In male germ cells, the repair of DNA double strand breaks (DSBs) is differently regulated than in somatic cells. During spermatogenesis, a small pool of stem cells produces spermatogonia that, after a series of clonal divisions, differentiate into spermatocytes. Spermatogonia are radiosensitive and probably use the non homologous end joining (NHEJ) pathway to repair DSBs, since the Ku70-deficient testis displays elevated levels of DSBs [1]. During meiotic prophase I, an efficient and proper meiotic homologous recombination repair of Spo11-induced DSBs is required for accurate chromosome segregation during metaphase I [2]. An interplay between homologous recombination (HR) and has been reported during the repair of ionizing irradiation (IR) induced DSB in early and late meiotic prophase cells [3]. However, the post-meiotic germ cells are very sensitive to genomic damage since their only available repair pathway, NHEJ, is known to be error-prone. As these cells develop to form mature sperm, they progressively lose the ability to repair DNA damage that may accumulate and be transmitted to the zygote and ultimately to the embryo [4]. In agreement with this, we have previously found that the repair capacity of round spermatids diminishes with ongoing development of these cells [5]. Moreover, sperm derived from spermatids that were irradiated early during their development displayed a higher frequency of chromosome aberrations (CA) in the fertilized egg than when derived from germ cells irradiated at the other spermatogenic stages [6,7]. A number of authors including us have extensively studied DNA damage signaling and repair mechanisms in different types of male germ cells up to the elongating spermatid stage [1,3,5,8,9,10,11]. The induction of DSBs and the DNA damage response elicited by IR has also been studied in hamster, rat, mouse and human elongating spermatids [11,12,13,14,15]. Nonetheless, the direct response of elongated spermatids to irradiation-induced DSBs and their DSBs repair capacity and thus, the role of the elongated spermatid stage in the genetic integrity of the male gamete is still poorly understood.

During spermiogenesis, histone-based chromatin structure is nearly completely substituted by a protamine-based one to induce doughnut-shaped supercoils that are more efficient for packing DNA into a small space than histone-based chromatin [16,17]. The chromatin remodeling steps are specifically associated with transient, endogenous DNA strand breaks that occur in developing mammalian spermatids [12,18,19]. DNA strand breaks have been observed in all elongating murine spermatids at Step 9 and decrease in number during Steps 10 and 11 [12]. Comet assay investigations under neutral conditions [20] show a clear accumulation of DSBs during spermatid elongation, which are eventually repaired during subsequent steps [18].

In response to DSBs, cells phosphorylate the histone H2 variant H2AX at serine 139, inducing the formation of nuclear foci of phosphorylated H2AX (then called γ-H2AX) at the sites of damage [21]. In the low dose range, each γ-H2AX focus represents a DSB when observed in non-S phase cell [22,23,24,25]. Hence, γ-H2AX has been extensively used as a marker of DSBs [3,5,8,9,23,24,26,27,28]. Besides γ-H2AX elongated spermatids express high levels of adenosine diphosphate ADP-ribose polymer and DNA polymerase activity during chromatin remodeling indicating that a physiological DSB repair response is triggered [14,19]. However, probably due to the compact size of elongated spermatids, the chromatin condensation [29] and the background of γ-H2AX chromatin staining at this stage, previous studies failed to show γ-H2AX foci in elongated spermatids in squash preparations or in testicular sections. Recently, neutral comet assay and flow cytometric analysis of γ-H2AX have shown no direct effects of IR on elongated spermatids after *in vivo* exposure to an X-ray dose of 4 Gy [11]. The authors failed to detect a DSBs response within the first two hours after irradiation. A subsequent long-term time course analysis revealed the appearance of DNA lesions in elongated spermatids between 27 and 45 days after irradiation, suggesting that irradiation of proliferating spermatogonia delivers a radiation insult that manifests at a later developmental stages, and according to Cordelli *et al.* [11] activates a process leading to DNA fragmentation in elongating spermatids. Therefore, investigating the DNA damage response during spermatid elongation in wild type mice and in mice deficient for NHEJ and HR is of interest.

Given their haploid character, spermatids must resolve exogenous and programmed DSBs by the classical or alternative NHEJ pathways. The classical non homologous end joining (cNHEJ) pathway relies on a set of proteins that recognize, bind and repair DSBs without homology. These proteins include DNA-PKcs which is recruited to the site of damage by the Ku70 and Ku80 proteins, followed by the recruitment of the ligation complex X-Ray Repair Cross-Complementing Protein 4 (XRCC4)/DNA ligase IV. The alternative route requires the synaptic activity of Poly Adenosine diphosphate (ADP)-Ribose Polymerase 1 (PARP1) in addition to the ligation activity of both X-Ray Repair Cross-Complementing Protein 1 (XRCC1) and DNA ligase III [30]. The kinetics of γ-H2AX loss in irradiated round spermatids and the expression analysis of NHEJ proteins in round spermatids revealed that both classical and alternative NHEJ pathways are active in this cell type [5]. However, it is not known whether these NHEJ pathways are active in the damage-sensitive elongated spermatids.

Here we have studied the expression of NHEJ proteins in mouse testis sections to address the question whether classical and alternative NHEJ pathways may be active to repair DSBs induced during the programmed DNA fragmentation during chromatin compaction in elongated spermatid nuclei. To this end, we quantified γ-H2AX foci present associated with chromatin remodeling and determined the kinetics of γ-H2AX foci formation and disappearance (repair) after γ irradiation, by studying elongated spermatids present in meiotic spread preparations from Rad54/Rad54B double knockout (dko) mice, DNA-PKcs deficient Severe Combined Immunodeficiency (SCID) mice, PARP1-inhibitor treated mice and their appropriate wild types. In addition, protein expression patterns were studied to see which NHEJ pathway is used by elongated spermatids during the chromatin remodeling process and after irradiation.

## 2. Results

### 2.1. Ku70 and 53BP1 Are Not Expressed in Elongated Spermatids

**Figure 1:**
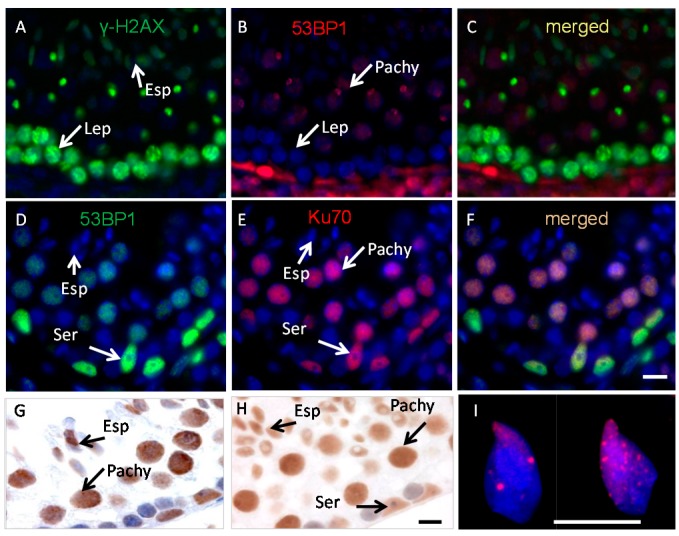
Localization of the phosphorylated-H2 variant (γ-H2AX, green) (**A**), 53 binding protein1 (53BP1, red, **B**,**C** and green, **D**), Ku70 (red, **E**,**F**), X-ray repair cross-complementing protein 1 (PARP1) (**G**) and X-ray repair cross-complementing protein1 (XRCC1) (**H**) in non-irradiated testis sections; (**I**) Meiotic spreads of elongated spermatids from RAD54/RAD54B deficient mice stained for γ-H2AX and counterstained with DAPI (blue). Elongated spermatids (Esp), Leptotene (Lep), Pachytene (Pachy), Sertoli (Ser). Scale bars: 10 µm.

Testis sections of wild-type mice were stained using antibodies against γ-H2AX, Ku70, 53BP1, PARP1 and XRCC1 to analyze the expression of these proteins during chromatin remodeling in elongated spermatids (Figure 1). Consistent with previous studies [14,19] γ-H2AX was found to be expressed at spermiogenesis Steps 9–12 of spermatid development (Stages X–XII; Stage X is shown, Figure 1A,C). Apart from an overall diffuse or dense staining we could not see γ-H2AX foci in elongated spermatids in sections before or after γ irradiation. However, staining meiotic spread preparations with the same antibody showed clear γ-H2AX foci in elongated spermatids (Figure 1I) before and 1 and 8 h after irradiation. No staining for Ku70 was found in spermatocytes in early meiotic prophase consistent with previous reports [31], indicating that the cNHEJ pathway is silent in this cell type. On the other hand, Ku70 was expressed in Sertoli cells, B spermatogonia, late spermatocytes and round spermatids, cell types in which the cNHEJ pathway is thought to be active (Figure 1E,F). However, there was no Ku70 protein in elongated spermatids at all steps of their development (Step 11/Stage XI is shown, Figure 1D,F).

No staining for 53BP1 was observed in elongated spermatids while 53BP1 showed an expression pattern similar to that of Ku70 in testicular cells including Sertoli cells, B spermatogonia, late spermatocytes and round spermatids (Figure 1B,D). Together, the data on Ku70 and 53BP1 indicate that in elongated spermatids some of the main components of the cNHEJ repair proteins are not expressed, or below the detection level. However, as in our earlier studies, both PARP1 and XRCC1 (Figure 1G,H respectively) were found to be expressed in elongated spermatids with no clear variation in the staining pattern before and after irradiation.

### 2.2. Incomplete Meiotic Recombination Repair in Late Spermatocytes Increases the Numbers of Background Foci during Spermatid Remodeling

**Figure 2:**
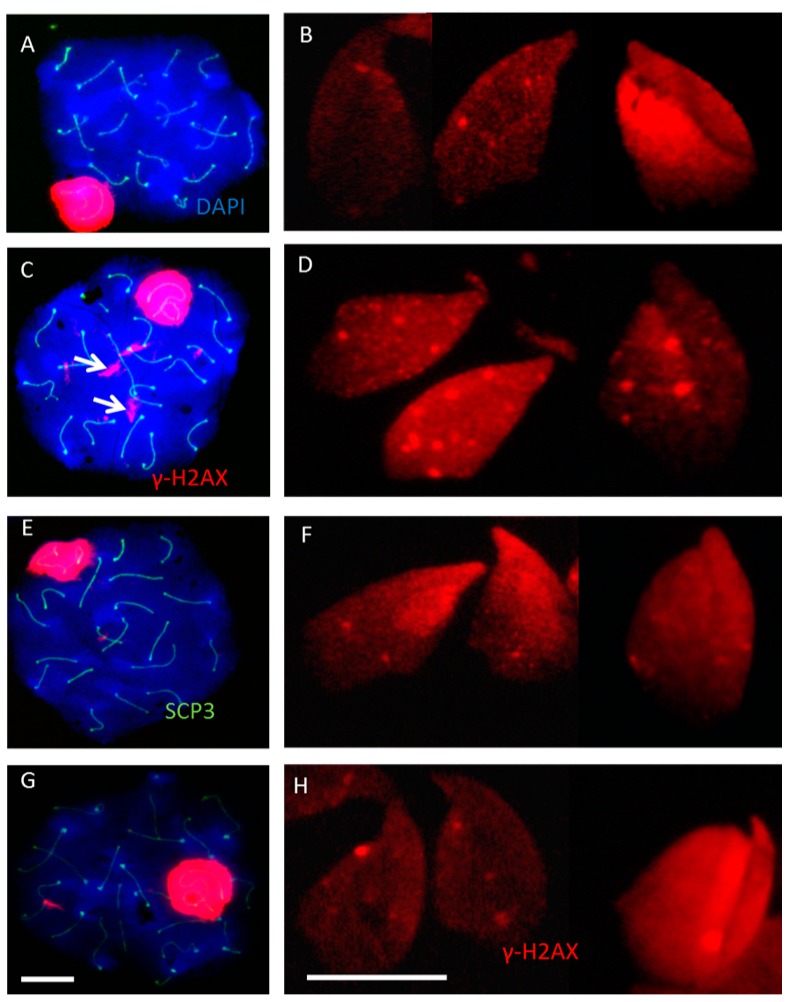
Representative images from non-irradiated meiotic spreads of pachytene spermatocytes and elongated spermatids stained for γ-H2AX (red) from wild type (**A**,**B**); RAD54/RAD54B double knockout mice (**C**,**D**); Severe Combined Immunodeficiency (SCID) mice (**E**,**F**) and PARP1 inhibitor (DPQ)-treated mice (**G**,**H**). In pachytene, spermatocytes of Rad54/Rad54B dko mice more large (L) foci can be seen (arrows, **C**), more foci in elongated spermatids are also shown in addition to the staining background (**D**). Scale bars: 10 µm.

**Figure 3:**
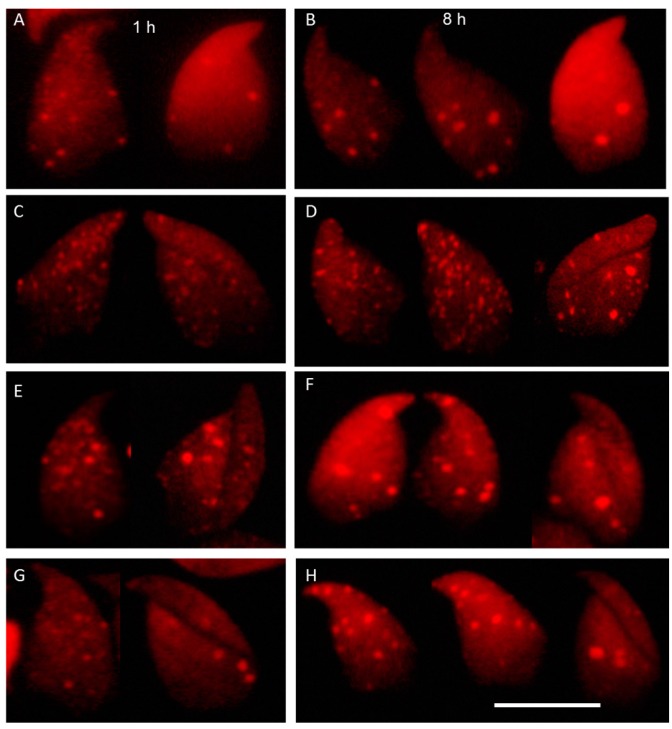
Representative images from irradiated meiotic spreads of pachytene spermatocytes and elongated spermatids stained for γ-H2AX, at 1 h and 8 h after ionized radiation (IR) in wild type (**A**,**B**); RAD54/RAD54B double knockout mice (**C**,**D**); SCID mice (**E**,**F**) and PARP1 inhibitor (DPQ)-treated mice (**G**,**H**). Scale bars: 10 µm.

In testis sections (Figure 1) and testis squash preparations [19], it was not possible to localize γ-H2AX foci in elongated spermatids, probably due to the high staining background and the more condensed chromatin and smaller size of nuclei as compared to detergent-spread preparations. Studying elongated spermatids in meiotic spread preparations from RAD54/RAD54B knockout mice and SCID mice, we detected the expected background staining of pan-chromatin staining in elongated spermatids (from Steps 9–12). In addition, we saw clear γ-H2AX foci (Figure 1I, Figure 2 and Figure 3) enabling their enumeration. A relatively high number of background foci were noted in elongated spermatids of RAD54/RAD54B knockout mice (Figure 2). Random quantification showed a 2–3-fold increase in the number of these foci in RAD54/RAD54B knockout mice compared to the wild type, SCID and PARP1-inhibited mice (Figure 3). Our previous analysis of γ-H2AX signals in non-irradiated RAD54/RAD54B deficient spermatocytes [3] indicated an incomplete meiotic recombination repair as evidenced by a pronounced increase in the numbers of γ-H2AX foci in late prophase primary spermatocytes. γ-H2AX foci numbers in elongated spermatids from SCID mice and PARP1-inhibited mice were comparable to those in wild type mice (Figure 2). Together these data suggest that incomplete meiotic recombination repair in RAD54/RAD54B-deficient spermatocytes increases the number of background foci during chromatin remodeling in elongated spermatids.

### 2.3. DNA Damage and Repair Responses to Irradiation-Induced DSBs in Elongated Spermatids

**Figure 4:**
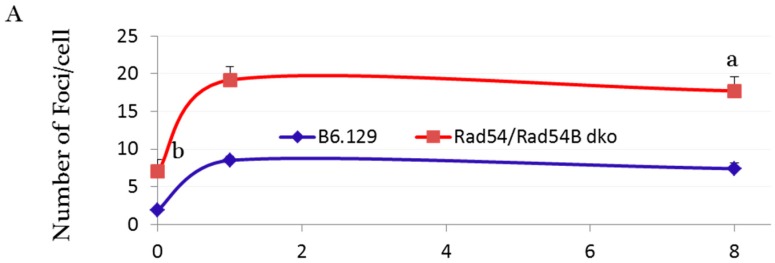

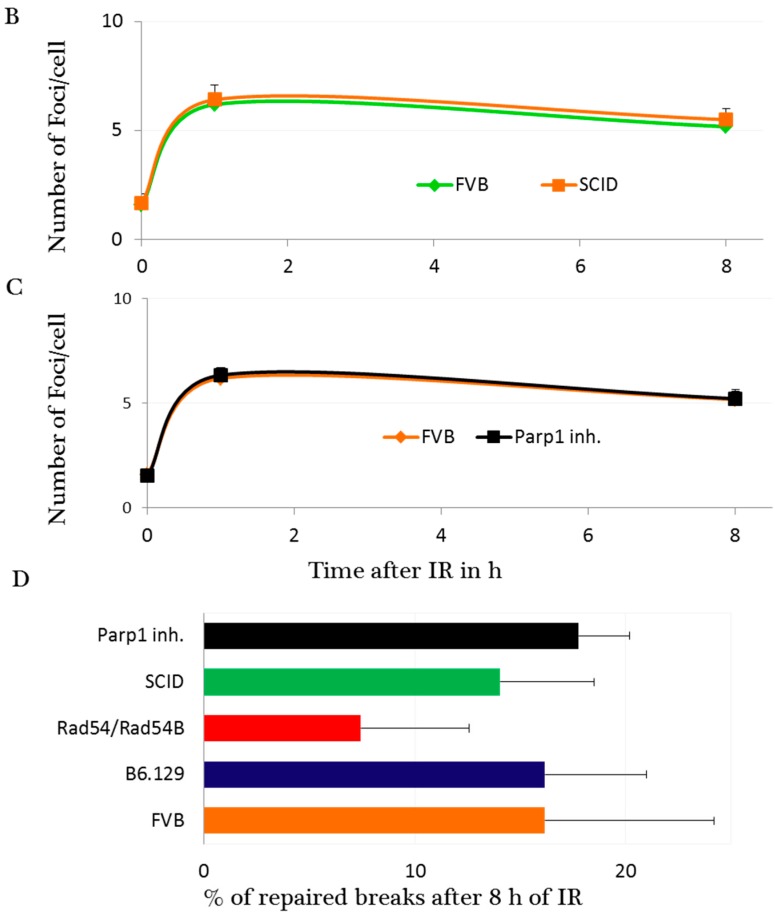
The kinetics of γ-H2AX foci formation and removal after IR in elongated spermatids. Number of γ-H2AX foci per cell in RAD54/RAD54B double knockout mice (**A**), SCID mice (**B**) and PARP1 inhibitor (DPQ)-treated mice (**C**) relative to the wild type mice. Between 40 and 50 cells were counted per mouse, data are presented as mean ± SEM, *n* = 3. *p* < 0.01, ^a^ compared to 8 h irradiated B6.129, ^b^ compared to the elongated spermatids in all non-irradiated mice. Percentages of repaired breaks after 8 h post- IR in wild type and other mice (**D**).

The absence of Ku70 and 53BP1 proteins in elongated spermatids indicates that classical NHEJ is not active during the chromatin remodeling steps (Figure 1). In contrast, PARP11 and XRCC1 are present in elongated spermatids verifying our staining results. However, that does not necessarily mean that these proteins do carry out repair of DSBs in elongated spermatids since the PARP1-XRCC1 complex is also involved in base excision repair. To clarify this point, we first checked the DSB formation and the DNA damage response in elongated spermatids by quantifying γ-H2AX foci numbers in elongated spermatids 1 and 8 h after γ irradiation. Significant IR-induced increases in foci numbers in irradiated elongated spermatids were found in all mice studied, indicating the induction of a DNA damage response (Figure 2 and Figure 4). Next, we estimated foci numbers in wild type, SCID and RAD54/RAD54B dko mice and in PARP1-inhibitor treated mice before and 1 and 8 h after irradiation. In wild type mice, between 1 and 8 h after IR, the numbers of γ-H2AX foci were only reduced by less than 18% in both the wild type mice (FVB and B6.129), indicating that elongated spermatids have a limited capacity to repair DSBs.

In elongated spermatids of Rad54/Rad54B-deficient mice, at 1 h after IR more irradiation-induced foci were observed than in wild type mice, probably indicating a higher radio-sensitivity. Furthermore, in these mutant mice at 8 h after IR no significant decrease in foci numbers was seen with the percentage of repaired breaks after 8 h of IR being 7.4% showing the absence of significant differences from their respective wild type control (B6.129). In DNA-PKcs-deficient SCID mice and PARP1-inhibited mice, the percentages of repaired breaks between 1 and 8 h after IR were around 14% and 18%, respectively. However, these percentages were not significantly different from the wild type, indicating a dispensable role for DNA-PKcs in the repair of the IR-induced DSBs in elongated spermatids. Foci appeared slightly larger in size at the 8 h time point compared to those at 1 h post irradiation (Figure 3), suggesting the presence of complex or irrepairable DSBs [32]. Moreover, except for the FVB wild type and PARP1-inhibited mice that showed a significant reduction in foci numbers after 8 h (*p* ≤ 0.02), all mice studied failed to show a significant difference between foci numbers at 1 and 8 h post IR. Taken together, the significant reduction in foci numbers 8 h after IR in FVB wild type and PARP1-inhibited mice; the low percentages of DSB foci reduction (≤18%) in all other mice studied, including the wild type and the PARP1-inhibited mice; and the insignificant differences in percentages of repaired breaks between the wild type and other phenotypes (Figure 4), indicate a limited repair capacity of IR-induced DSBs in elongated spermatids.

## 3. Discussion

Here, we analyzed the expression pattern of the NHEJ repair proteins in elongated spermatids to study DSB formation and repair in a fragile stage of chromatin remodeling that occur during spermatid elongation. The detection of γ-H2AX DSB foci in elongated nuclei of spermatids in detergent-treated meiotic spreads enabled us to compare the background and radiation-induced levels of DSB foci in elongated spermatids from different mouse lines; *i.e.*, RAD54/RAD54B dko, SCID, PARP1 inhibitor (DPQ)-treated mice and their appropriate wild type controls. Our data indicate that while PARP1 and XRCC1 proteins were readily detected, Ku70 and 53BP1 proteins were not expressed during spermatid elongation, while γ-H2AX foci formed after IR revealing a direct DNA damage response to the irradiation-induced DSBs in elongated spermatids.

The analysis of the expression pattern of the main proteins of the NHEJ pathway in the non-irradiated and irradiated testis showed that Ku70 and 53BP1 are not expressed in elongated spermatids. During the classical pathway of NHEJ, the Ku proteins recruit DNA-PKcs to the site of damage. Subsequently, both end-positioned Ku and DNA-PKcs mediate the recruitment of the XRCC4/DNA ligase IV complex that is responsible for the ligation step. Also 53BP1 stimulates the end-joining reaction by DNA ligase IV/Xrcc4 *in vitro* [33] and plays a major role in NHEJ repair *in vivo* [8,34]. In the mouse testis 53BP1 and K70 show a similar expression pattern in testicular cell types, both are down regulated during early meiotic prophase where repair of Spo11 induced DSBs should be carried out by HR repair [31]. 53BP1 and Ku70 are expressed in Sertoli cells and in late meiotic prophase I spermatocytes as well as in haploid round spermatids where NHEJ pathway is considered to be the only possible repair pathway [3,5]. Earlier studies by our group have shown that the main proteins of NHEJ DNA-PKcs and Ku (Ku70 and Ku86) are not expressed in elongated spermatids [35] suggesting that the classical pathway is not involved in DSB repair in this cell type.

However, we found both PARP1 and XRCC1 to be expressed in elongated spermatids in Stages X–XII, but without a clear response to ionizing irradiation. Both these proteins are constituents of an alternative NHEJ pathway that requires a recently detected synaptic activity of PARP1 and the ligation activity of the XRCC1-DNA ligase III complex [30]. Besides this, both proteins are also involved in base excision repair. Interestingly, using hamster cell lines, Mansour *et al.*, [36] have shown that Ku is the main regulator for the switching between the classical-NHEJ and the alternative NHEJ pathway, the latter being activated when Ku is absent. So could the alternative NHEJ pathway indeed be active in elongated spermatids where Ku70 and Ku86 are not expressed? In round spermatids the repair capacity after IR is reduced with the ongoing development of these cells. Then, PARP1 inhibition only induces a significant increase in persistent γ-H2AX foci in early round spermatids in epithelial Stages I–III but not in subsequent developmental steps of round spermatids [5]. Therefore, being expressed in elongated spermatids does not mean that PARP1 and XRCC1 carry out repair of IR-induced DSBs in this cell type. Indeed our finding that inhibition of PARP1 has no effect on the repair of IR-induced DSBs in elongated spermatids suggests that the alternative NHEJ pathway is not active in elongated spermatids. However, although the background level of foci in PARP1-inhibited elongated spermatids does not significantly differ from that in the wild type, PARP1 inhibition may not be the appropriate model to clarify the role of the alternative pathway during chromatin remodeling. Further studies in this direction are required.

In view of the immunocytochemical localization of (poly ADP-ribose) polymer (PAR), the indicator of PARP activity, in round and elongating human spermatids [37] Meyer-Ficca *et al.* [14] hypothesized that the expression of γ-H2AX and the formation of PAR in elongated spermatids during the chromatin remodeling steps may facilitate DNA strand-break management during spermatid maturation. However, sperm from *PARP-1*^−/−^ mice only show a subtle loss of nuclear elongation and motility without an impact on fertility [15]. Thus, PARP1 does not significantly contributes to the repair of IR-induced DSBs in elongated spermatids, and perhaps much of the irradiation-induced damage in elongated spermatids will be passed on to the sperm and then to the zygote and possibly the embryo (see below). However, DNA damage that reaches the zygote will be subject of DNA repair after restoration of nucleosomal chromatin structure in the male pronucleus. Currently, there is no evidence for a direct involvement of the alternative NHEJ pathway in the repair of transient DSBs during chromatin remodeling and the DSB repair efficiency beyond Step 13 of spermiogenesis remains to be determined. Chromatin structure is considered a prime candidate for the modulation of alternative-NHEJ since parameters other than chromatin acetylation may influence alternative NHEJ efficiency [38]. During spermatid elongation the chromatin structure is changed from a nucleosomal one to a lariate structure by first transition proteins and eventually protamines replacing most histones [39,40]. Therefore, the γ-H2AX signal may diminish once histone replacement is nearly completed and that the change to a tightly compacted chromatin structure is influencing the efficiency of DNA repair. Therefore, it may be that this change in chromatin structure is influencing the efficiency of DNA repair.

In somatic cells, the repair of radiation-induced DSBs is immediately initiated and most DSBs are repaired within the first few hours after exposure. However, the repair of DSBs in male germ cells is slower and differently regulated [8]. In the present study, significant increases in IR-induced foci numbers were noted in spread preparations of elongated spermatids at 1 and 8 h after IR. In contrast, flow cytometry analysis of γ-H2AX in X-irradiated elongated spermatids did not reveal a direct damage response at 2 h after IR [11]. This likely relates to preparation differences in the two studies—surface spreading with detergent and flow cytometry analysis, with the latter being less sensitive in the low dose range and the presence of γH2AX background signals that usually occur during nuclear elongation. In agreement, *in situ* quantification of γ-H2AX foci in elongated spermatid from meiotic spreads clearly showed a DSB damage response in elongated murine spermatids with limited repair capacity. In a study with hamster elongated spermatids where IR-induced DNA damage was detected by alkaline elution, a low induction frequency of single strand breaks and base damage was noted [41]. In this case too, hardly any repair was found. This agrees with radiation inducing a delayed (after 27 days) DNA fragmentation and apoptosis-like processes during spermatid elongation [11]. The authors stated that it is difficult to understand how the damage persisted from the earlier stages of meiosis till the spermatid stage in view of the multiple cell cycles and checkpoints during germ cell development. Therefore, the authors proposed that spermatogonia and spermatocytes in early meiotic prophase retain a memory of the radiation insult, which activates a process leading to a delayed DNA fragmentation in elongated spermatids and also in epididymal spermatozoa [11]. Spermatogonia and late spermatocytes use the error prone NHEJ to repair the IR-induced breaks [1,3,31] rendering it likely that a high dose of 4 Gy [11] will probably lead to inefficient or mis-repaired breaks. Consistent with that, spermatozoa derived from irradiated pre-meiotic germ cells were found to carry DNA strand breaks [42,43,44,45]. Also sperm DNA fragmentation is more frequent in infertile men, sperm of fertile men display DNA fragmentation but to a lesser extent [46,47,48,49]. Therefore, physiological DNA strand breaks present in mature spermatozoa and such derived from IR of earlier germ cell types were proposed to derive from an active process of DNA fragmentation occurring during meiosis or spermiogenesis [11].

Comparing γ-H2AX foci numbers in elongated spermatids in meiotic spread preparations of RAD54/RAD54B double knockout mice, SCID mice, PARP1 inhibitor (DPQ)-treated mice and their appropriate wild-type controls, revealed 2–3-fold higher foci numbers in RAD54/RAD54B knockout mice than in all other genotypes. γ-H2AX foci in elongated spermatids are related to transient DNA DSBs that occur during chromatin remodeling during meiosis or spermiogenesis [11,12,50]. Possibly, the higher number of γ-H2AX foci during chromatin remodeling in RAD54/RAD54B knockout mice are related to an earlier defect during mutant spermatogenesis. However, although both RAD54/RAD54B dko and SCID males are fully fertile and litter size is normal [51], the presence of persistent large (L) γ-H2AX foci in late spermatocytes of non-irradiated RAD54/RAD54B knockout mouse and the RAD51 deposits observed by Wesoly *et al.* [51] point towards the incomplete meiotic recombination repair [3]. Seminiferous tubules of RAD54/RAD54B knockout mice show no increase in apoptosis [35,51] suggesting that meiotic Stage IV and spindle checkpoints are not activated [52], which probably lead to transmission of HR generated damage to haploid cells. In agreement, some large and small γ-H2AX foci were detected in round spermatids of non-irradiated RAD54/RAD54B deficient mice but not in round spermatids from SCID or PARP1-inhibited mice [3], which aligns with fertility in the *PARP1*^−/−^ mouse [53] and similar numbers of γ-H2AX-positive spermatids and spermatocytes in *PARP1*^−/−^ and wild type mice. In all, we have shown that spermatid elongation goes along with the reduced fidelity of NHEJ that may translate in a particular vulnerability of this differentiation stage to genotoxic insults.

## 4. Materials and Methods

### 4.1. Animals, Irradiation and Fixation

Male DNA-PKcs deficient SCID mice (7–8 weeks of age; Charles River, Maastricht, The Netherlands) and their wild type control, B6.129 synthetic mice (2–4 months of age, kindly provided by Peter de Boer), were used. The latter have been described by Wesoly *et al.* [51] and Derijck *et al.* [54]. To study the effect of PARP1 inhibition on DNA DSBs repair, male FVB mice (8–10 week of age, Charles River, Maastricht, The Netherlands) were used. Mice either received injections of the PARP1 inhibitor, 3,4-dihydro-5-[4-(1-piperidinyl)butoxy]-1(2H)-isoquinolinone, DPQ (D5314 Sigma, St. Louis, MO, USA, 10 mg/kg of body weight) dissolved in dimethyl sulfoxide (DMSO) or DMSO alone, 1 h before γ irradiation (IR) and 3.5 h later (for the 8 h time point). Mice were either sham-irradiated (4 mice per group) or exposed to a whole body dose of 1 Gy of 6 MV γ rays (91 MU, Elektra, Crawley, UK). At this concentration PAP1 inhibition has been shown to be effective *in vivo* [5,55] Irradiated mice were sacrificed at 1 or 8 h after irradiation. Mice were killed by CO_2_ asphyxiation. One testis was fixed in 4% paraformaldehyde in PBS for 24 h at 4 °C and from the other testis nuclear spreads of spermatogenic cells were prepared (see below). Tissues were washed in 70% EtOH prior to embedding in paraffin (Stemcowax; Adamas Instruments, Amerongen, The Netherlands). The animals were used and maintained according to the regulations provided by the animal ethical committee (Dierexperimenten Commissie, DEC) of Utrecht University that also approved the experiments. The project code of the project is DEC-nr-2007.III.07.097, PARP1 inhibitor treatment mice approved in July 2008.

### 4.2. Immunohistochemistry

Testis sections (5 m) of irradiated or sham-irradiated mice were mounted together on TESPA (3-aminoproyl-triethoxysilane)-coated glass slides and dried overnight at 37 °C. Sections were dewaxed in xylene and hydrated in a graded series of alcohols. For PARP1, and XRCC1 staining, sections were boiled twice for 10 min in 0.01 M sodium citrate using a microwave oven (H2500; Bio-Rad, Hercules, CA, USA). Sections were incubated in 0.35% H_2_O_2_ in PBS for 10 min. Blocking was done in 5% BSA (Sigma, A-7906)/5% goat serum (Vector Laboratories, S-1000, Burlingame, CA, USA) in PBS. The primary antibodies were: pre-diluted mouse monoclonal anti-XRCC1 [5] (1:5, Abcam ab54393, Cambridge, UK) and rabbit polyclonal anti-PARP1 [5] (1:200, Abcam, ab2168.500, Cambridge, UK). The slides were washed in PBS and then incubated with the secondary antibody, PowerVision Poly Hrp anti-mouse/rabbit/rat (ImmunoVision Technologies, Co., Brisbane, CA, USA), ready to use, for 40 min at room temperature. Bound antibodies were visualized using 0.3 g/L 3,3-diaminobenzidine (DAB, Sigma) in PBS, to which 0.03% H_2_O_2_ was added. Sections were counterstained with Mayer hematoxylin. Sections were dehydrated in a series of graded alcohols and xylene and mounted with Pertex (Cellpath Ltd., Hemel Hempstead, UK).

### 4.3. Immunofluorescence

For immunofluorescence, slides were washed in PBS, incubated for 10 min in PBS including 0.04% Triton X and then incubated with blocking solution (10% goat serum and 10% BSA in PBS). Slides were incubated with mouse monoclonal anti-phospho-H2AX (Ser139) [8] (1:200, JBW-301,05-636, Upstate Biotechnology, Lake Placid, NY, USA), goat anti-Ku70 [1] (sc-1487, 1:10, Santa Cruz Biotechnology, Santa Cruz, CA, USA) and anti 53BP1 rabbit polyclonal [1] (1:400). The secondary antibodies Donkey anti-goat (Santa Cruz Biotechnology), goat anti-rabbit (Alexafluor 488, A-11008), goat anti-mouse (Alexafluor 488, A-21121 and 594, A-21125) and Texas Red-labeled goat anti-mouse, were obtained from Jackson ImmunoResearch (West Grove, PA, USA) and were applied at a dilution of 1:1000. The slides were incubated with DAPI (0.5 g/mL for 10 min), mounted in VECTAshield (Vector Lab., H-1000) and viewed with an Olympus AX70 microscope (Olympus Optical Co., Ltd., Tokyo, Japan). Images were recorded digitally. All antibodies were tested in individual staining reactions for their specificity and performance. Controls without primary antibodies were all negative (not shown).

### 4.4. Surface-Spread Preparations

Nucleus spreads were made as previously described [3,56]. Briefly, a suspension of spermatogenic cells in MEM was obtained and then incubated with a hypotonic buffer (17 mM sodium citrate, 50 mM sucrose, 30 mM Tris HCl, pH 8.2). After centrifugation, the pellet was carefully resuspended in a 100 mM sucrose solution and applied over a PFA-coated glass slide (1% PFA, 0.15% Triton X-100, pH 9.2–9.5). The slides were kept in a humidified atmosphere in a box to slow down drying out. After 1.5 h the box was opened and the slides were washed in 0.08% photo-flo (Sigma P7417).

### 4.5. The Kinetics of γ-H2AX Foci Loss after Irradiation

Numbers of γ-H2AX foci were counted in elongated spermatids in nucleus spreads prepared from Rad54/Rad54B knockout mice, SCID mice, PARP1-inhibited FVB mice and in the wild type FVB and B6.129 mice. Foci before and after irradiation (at 1 and 8 h) were quantified in Steps 9–13, where γ-H2AX was suggested to be expressed [19].

### 4.6. Statistical Analysis

Statistical analysis between groups was done by one way analysis of variance (Dunnett’s Multiple Comparison Test) using GraphPad software (GraphPad Software, San Diego, CA, USA).

## 5. Conclusions

In all, our data show that the NHEJ repair pathway is not available for elongated spermatids to repair the programmed and the IR-induced DSBs, which likely underlies the limited repair capacity of these cells. However, although elongated spermatids express the alternative NHEJ proteins, PARP1-inhibition has no effect on the repair kinetics after IR, suggesting that the damage may be passed to the sperm. Finally, our genetic mutant analysis suggests that an incomplete or defective meiotic recombination of Spo11-induced DSBs may lead to carryover of DSB damage or induce a delayed fragmentation during the sensitive stage of programmed chromatin remodeling in elongated spermatids.
